# Effects of a structured SBIRT training program for hospital nursing leaders on utilization of SBIRT within their medical-surgical units: cohort study

**DOI:** 10.1186/s12912-025-03079-9

**Published:** 2025-04-23

**Authors:** Robin Newhouse, Jon Agley, Giorgos Bakoyannis, Melora Ferren, C. Daniel Mullins, Alyson Keen, Erik Parker

**Affiliations:** 1 Indiana University School of Nursing, Indiana University, 600 Barnhill Drive, NU 132, Indianapolis, IN 46202 USA; 2 Prevention Insights and Department of Applied Health Science, Indiana University School of Public Health - Bloomington, Indiana University Bloomington, 809 E. 9th St, Bloomington, IN 47408 USA; 3 Indiana University Fairbanks School of Public Health and Indiana University School of Medicine, 410 West 10th St., Suite 3000, Indianapolis, IN 46202 USA; 4 Indiana University Health, Fairbanks Hall, 340 West 10th St, Indianapolis, IN 46202 USA; 5 University of Maryland School of Pharmacy, Saratoga Building, 12th Floor 220 Arch Street, Baltimore, MD 21201 USA; 6 Interprofessional Evidence-Based Practice and Research, Indiana University Health, 1701 N. Senate Ave, Indianapolis, IN 46202 USA; 7 Biostatistics Consulting Center, Indiana University School of Public Health – Bloomington, Indiana University Bloomington, Bloomington, IN USA

**Keywords:** Nurse, Nursing, Screening, Brief intervention, Referral to treatment, SBIRT, Hospital, Quality, Cohort, Substance use

## Abstract

**Background:**

Psychoactive substances contribute to numerous deaths annually, and more than 60% of the US population aged 12 + years reports past-month substance use. Screening, brief intervention, and referral to treatment (SBIRT) may support identification of substance-related risks and facilitate targeted interventions, but best practices and implementation designs remain elusive. Our study examined whether a standardized SBIRT toolkit and training-of-trainers for nurse site coordinators was prospectively associated with documented performance of core SBIRT-related functions in medical-surgical hospital units.

**Methods:**

This was a prospective cohort study conducted from January 2018 to May 2019 in 14 adult medical-surgical units (one/hospital). Hospitals were randomly allocated to two groups (*n* = 7 hospitals/each), which received identical interventions: an SBIRT training-of-trainers (8 h), supportive follow-up, and a toolkit containing information, resources, and guidance. However, group 1 sites were trained four months earlier than group 2 sites. At three points (baseline, 10-months, and 16-months), 61 patient records per hospital unit (*n* = 854) were randomly selected for extraction. Inclusion criteria for random selection were age (18+) and being admitted and discharged from the selected unit. Main outcome measures were analyzed using generalized linear mixed models, including screening within 24 h of admission, using a validated screening tool, screening positive, and receiving a brief intervention or referral to treatment.

**Results:**

For groups 1 and 2, patients had 1.81 and 2.66 greater odds, respectively, of being screened for alcohol at 10-months, 1.92 and 4.68 greater odds of being screened for drugs, and 1.96 and 2.06 greater odds of being screened for tobacco. For hospital group 2, patients also had greater odds of being screened for alcohol (3.92), drugs (6.31), and tobacco (2.41) at 16-months. For both hospital groups and benchmarks, patients were hundreds of times more likely to be screened with a validated tool, reflecting a shift from near absence of such behaviors (around 1% prevalence) to prevalence rates from 24 to 56%.

**Conclusions:**

The SBIRT intervention was associated with the initiation and sustained use of validated screening tools for alcohol and drugs, and with short-term increases in overall alcohol, tobacco, and drug screening prevalence.

**Trial registration:**

ClinicalTrials.gov NCT03560076.

## Background

In the United States (US), psychoactive substance use contributes to hundreds of thousands of deaths annually [1–3], and nearly 60% of the US population aged 12 + years reports past-month substance use [4]. Researchers and clinicians have described multiple categories and patterns of substance use, including light or infrequent use, disordered use (resulting in a clinical diagnosis), or harmful subclinical patterns of use [5–9]. Offering appropriate clinical services often requires healthcare providers to understand their patients’ substance use – which is one reason that the US Preventive Services Task Force (USPSTF) recommends screening adults for drug use (B grade) and adolescents and adults for alcohol use (B grade), and asking adults about tobacco use (A grade) [10]. 

### Screening, Brief Intervention, and Referral to Treatment (SBIRT)

SBIRT is a multi-component approach designed to identify risky substance use and provide one or more interventions based on observed risk severity (such as a brief motivational conversation or a referral to a specialized treatment provider) [11]. The evidence basis for SBIRT is complex [12]. For alcohol, screening scores may vary by clinical setting (outpatient vs. inpatient) [13], brief intervention efficacy may vary by severity of alcohol use [14, 15], and there is debate about whether and how brief interventions facilitate patients’ use of treatment services [16, 17]. Important randomized studies continue to be conducted [18, 19]. The literature on other psychoactive substances is likewise complicated (we cite a small sample of such papers here [20–26]). Tobacco is an exception, for which multiple different types of interventions and referrals appear to support some level of smoking cessation [27–29]. 

Some of the complexity in the SBIRT literature may stem from high levels of study and intervention heterogeneity. SBIRT implementation research long has identified barriers to uptake (e.g., competing priorities, lack of time, insufficient training) [30–32], and researchers have suggested that tailoring procedures to the environmental context [33] and allowing adaptation of the intervention during preparation and implementation phases [32] may attenuate some barriers.

### The present study

We developed a scalable, standardized “toolkit” intervention with both fixed and modifiable components [12]. Numerous ERIC (Expert Recommendations for Implementing Change) strategies were incorporated into the intervention procedures (e.g., regular audits and feedback, developing academic partnerships, promoting adaptability, and multiple others) [34]. For this study, our goal was to test whether the standardized toolkit intervention increased completion of key SBIRT outcomes in inpatient medical wards [12]. These included: (a) screening for alcohol and drug use [10]; (b) using validated screening tools (alcohol and drugs), because the meaning of the word “screening” varies and is often treated as interchangeable with “asking” [35, 36]; (c) asking about tobacco use using direct questioning (e.g., yes/no [37]); and (d) provision of risk-appropriate services, including brief interventions for lower risk levels and referrals to treatment for higher risk levels [37]. 

We measured documented completion of SBIRT outcomes in 14 hospitals within a single healthcare system at three points (baseline, 10 months, and 16 months post-baseline). This study was originally designed as a waitlist cluster randomized trial, as preregistered (NCT03560076) and described in Newhouse et al. [12] However, due to timing issues with intervention implementation arising from the realities of hospital operation, the study - as conducted - was a prospective cohort study of two randomly allocated clusters of hospitals (*n* = 7 hospitals each) *without* the ability to conduct between-group comparisons.

## Methods

### Study design

**Fig. 1:**
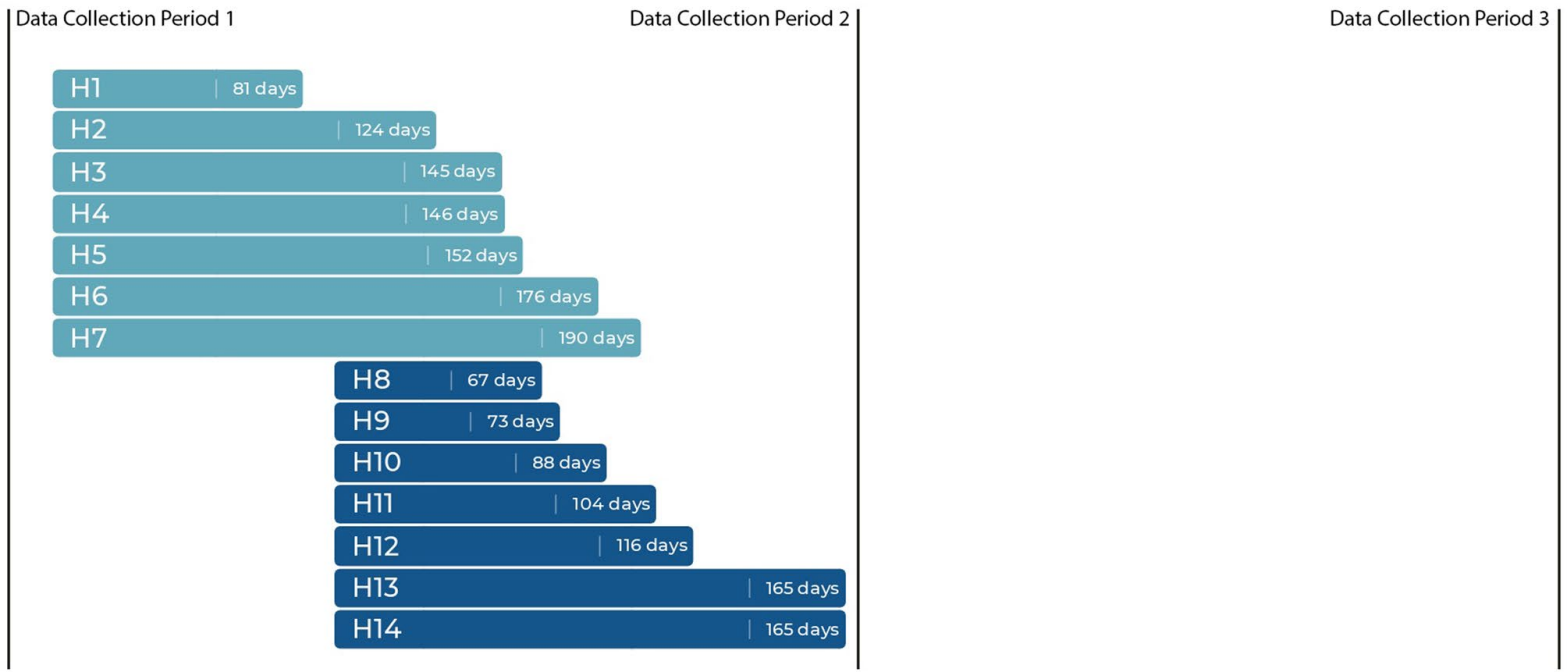
Relative data collection points and time from training to implementation (in days) for hospitals

This study tested for changes in primary outcomes contrasting baseline data and data collected at two time points after the intervention, and we report the results according to the Strengthening the Reporting of Observational Studies in Epidemiology (STROBE) statement for cohort studies [38]. The cohort timeline is shown in Fig. 1. The study was administratively reviewed by the Indiana University Institutional Review Board (#1801646970).

### Setting and participants

This study was conducted within a large healthcare system in the Midwestern US. Hospitals with adult medical-surgical units (*n* = 14) were considered eligible for inclusion. Executive leaders at each eligible facility approved study participation and selected one medical-surgical unit within their hospital to participate (see Table 1).

**Table 1:** Hospital characteristics by type (*N* = 14)

	*N*	Bed range	Mean beds (standard deviation)
Academic health centers	4	38–858	413 (337.73)
Community hospitals	4	127–375	214 (110.79)
Critical access hospitals	6	15–25	23 (4.08)

We followed these 14 hospitals (in 2 groups of 7) over 18 months, from January 2018 through May 2019. The first group received the intervention in January 2018, and the second group received the same intervention in April 2018. Data collection for primary outcomes was conducted at baseline (January 2018), 10-month follow-up (October 2018), and 16-month follow-up (May 2019). All hospitals had confirmed SBIRT implementation prior to 10-month follow-up.

Due to the original cluster randomization design, hospitals received the same intervention but in two different groups. However, all hospitals had fully implemented the intervention prior to follow-up data collection, and qualitative data suggest that intervention and control hospitals were communicating about the intervention. As a result, between-group comparisons are inappropriate to conduct or interpret because observed differences between groups cannot be clearly attributed to the intervention.

### Intervention

The SBIRT intervention was a guided training-of-trainers (TOT) implementation process that included a single, formal training, supportive follow-up, and an extensive toolkit containing information, resources, and guidance on SBIRT and motivational interviewing (MI). The direct recipients of the intervention were registered nurse site coordinators within the medical-surgical unit at each hospital (*n* = 14). These coordinators were selected by hospital executives at each corresponding hospital after allocation of the hospitals to study group.

The site coordinators participated in an 8-hour standardized SBIRT implementation TOT session. There were 2 separate sessions, and coordinators either attended at baseline (group 1) or 4 months later (group 2). Training content from the group sessions is described in greater detail elsewhere [39]. Due to scheduling issues, 1 coordinator per group received individualized training. This represents a deviation from planned study protocol [12], but every effort was taken to ensure that the individual TOT was equivalent to the group session, and the same materials were provided.

We provided an investigator-developed toolkit to all coordinators when they received the TOT. The toolkit included materials for staff training, assessment, engagement, communication, planning, and evaluation [40]. Investigators and site coordinators were permitted to modify and update toolkits for their sites throughout the project to allow contextual tailoring to study materials. This approach was commonly observed in a scoping review of healthcare based SBIRT implementation studies [41]. 

Each site coordinator assessed the baseline organizational capacity at their facility and developed a plan for implementation specific to their facility’s needs and resources. The clinical expectations of participating medical-surgical units were considered core components and were not permitted to vary. However, all implementation strategies articulated in the toolkit procedures were permitted to be tailored to the unit context. For example, some sites opted for respiratory therapists to conduct tobacco brief interventions, while others identified one or more trained registered nurses (RNs) [42]. Site coordinators trained local facility staff on SBIRT, led implementation efforts, and described their SBIRT process upon initiation of the study [43]. The site coordinators and investigators met monthly to discuss progress and share knowledge.

### Variables and data sources

The primary outcome variables were the prespecified indicators that key components of SBIRT were used within the hospital unit, including screening, screening with a validated tool, screening ‘positive,’ receiving a brief intervention, and receiving a referral to treatment, separately for alcohol, other drugs, and tobacco. These elements were extracted from individual, randomly selected electronic health records (these variables and possible data states for each variable are shown in Table 2). However, we did not collect separate indicators for ‘screening’ and ‘screening using a validated tool’ for tobacco (in contrast to how we measured procedures for alcohol and other drugs) because a basic yes/no question is a validated approach to screen for tobacco (e.g., established clinical nursing practices like “Ask, Advise, Refer” for tobacco use a yes/no question) [44]. 

**Table 2:** Descriptive statistics separated by training cohort

	Group 1 Baseline *N* (%)	Group 2 Baseline *N* (%)	Group 1 10-Month *N* (%)	Group 2 10-Month *N* (%)	Group 1 16-Month *N* (%)	Group 2 16-Month *N* (%)
Patient screened within 24 h of admission (Alcohol)						
No	168 (39.3)	104 (24.4)	119 (27.9)	64 (15.0)	157 (36.8)	51 (11.9)
Yes	258 (60.4)	323 (75.6)	308 (72.1)	363 (85.0)	270 (63.2)	375 (87.8)
Missing	1 (0.2)	0 (0.0)	0 (0.0)	0 (0.0)	0 (0.0)	1 (0.2)
Patient screened using a validated tool (Alcohol)						
No	424 (99.3)	422 (98.8)	294 (68.9)	205 (48.0)	321 (75.2)	188 (44.0)
Yes	3 (0.7)	5 (1.2)	133 (31.1)	222 (52.0)	106 (24.8)	239 (56.0)
Missing	0 (0.0)	0 (0.0)	0 (0.0)	0 (0.0)	0 (0.0)	0 (0.0)
Patient screened positive (Alcohol)						
No	3 (0.7)	5 (1.2)	126 (29.5)	213 (49.9)	103 (24.1)	230 (53.9)
Yes	0 (0.0)	0 (0.0)	7 (1.6)	9 (2.1)	3 (0.7)	9 (2.1)
Missing	424 (99.3)	422 (98.8)	294 (68.9)	205 (48.0)	321 (24.8)	188 (44.0)
Patient received brief intervention (Alcohol)						
Received	0 (0.0)	0 (0.0)	6 (1.4)	7 (1.6)	2 (0.7)	5 (1.2)
Refused	0 (0.0)	0 (0.0)	1 (0.2)	0 (0.0)	1 (0.2)	0 (0.0)
Did not receive	0 (0.0)	0 (0.0)	0 (0.0)	1 (0.2)	0 (0.0)	0 (0.0)
Unable to determine	0 (0.0)	0 (0.0)	0 (0.0)	1 (0.2)	0 (0.0)	4 (0.9)
Contraindication	0 (0.0)	0 (0.0)	0 (0.0)	0 (0.0)	0 (0.0)	0 (0.0)
Missing	427 (100.0)	427 (100.0)	420 (98.4)	418 (97.9)	424 (99.3)	418 (97.9)
Patient received referral to treatment (Alcohol)						
Received	0 (0.0)	0 (0.0)	0 (0.0)	1 (0.2)	0 (0.0)	0 (0.0)
Refused	0 (0.0)	0 (0.0)	2 (0.5)	3 (0.7)	0 (0.0)	0 (0.0)
Did not receive	0 (0.0)	0 (0.0)	1 (0.2)	2 (0.5)	0 (0.0)	0 (0.0)
Unable to determine	0 (0.0)	0 (0.0)	3 (0.7)	1 (0.2)	2 (0.5)	5 (1.2)
Rec’d but no appointment scheduled	0 (0.0)	0 (0.0)	1 (0.2)	2 (0.5)	1 (0.2)	4 (0.9)
Missing	427 (100.0)	427 (100.0)	420 (98.4)	418 (97.9)	424 (99.3)	418 (97.9)
Patient screened within 24 h of admission (Drugs)						
No	191 (44.7)	150 (35.1)	132 (30.9)	67 (15.7)	170 (39.8)	56 (13.1)
Yes	236 (55.3)	277 (64.9)	295 (69.1)	360 (84.3)	257 (60.2)	371 (86.9)
Missing	0 (0.0)	0 (0.0)	0 (0.0)	0 (0.0)	0 (0.0)	0 (0.0)
Patient screened using a validated tool (Drugs)						
No	426 (99.8)	425 (99.5)	294 (68.9)	239 (56.0)	323 (75.6)	190 (44.5)
Yes	1 (0.2)	2 (0.5)	133 (31.1)	188 (44.0)	104 (24.4)	237 (55.5)
Missing	0 (0.0)	0 (0.0)	0 (0.0)	0 (0.0)	0 (0.0)	0 (0.0)
Patient screened positive (Drugs)						
No	1 (0.2)	2 (0.5)	127 (29.7)	178 (41.7)	97 (22.7)	227 (53.2)
Yes	0 (0.0)	0 (0.0)	6 (1.4)	10 (2.3)	7 (1.6)	10 (2.3)
Missing	426 (99.8)	425 (99.5)	294 (68.9)	239 (56.0)	323 (75.6)	190 (44.5)
Patient received brief intervention (Drugs)						
Received	0 (0.0)	0 (0.0)	3 (0.7)	8 (1.9)	5 (1.2)	5 (1.2)
Refused	0 (0.0)	0 (0.0)	1 (0.2)	0 (0.0)	0 (0.0)	1 (0.2)
Did not receive	0 (0.0)	0 (0.0)	0 (0.0)	0 (0.0)	1 (0.2)	1 (0.2)
Unable to determine	0 (0.0)	0 (0.0)	2 (0.5)	2 (0.5)	1 (0.2)	3 (0.7)
Contraindication	0 (0.0)	0 (0.0)	0 (0.0)	0 (0.0)	0 (0.0)	0 (0.0)
Missing	427 (100.0)	427 (100.0)	421 (98.6)	417 (97.7)	420 (98.4)	417 (97.7)
Patient received referral to treatment (Drugs)						
Received	0 (0.0)	0 (0.0)	0 (0.0)	0 (0.0)	0 (0.0)	0 (0.0)
Refused	0 (0.0)	0 (0.0)	1 (0.2)	1 (0.2)	1 (0.2)	0 (0.0)
Did not receive	0 (0.0)	0 (0.0)	0 (0.0)	3 (0.7)	0 (0.0)	1 (0.2)
Unable to determine	0 (0.0)	0 (0.0)	3 (0.7)	2 (0.5)	4 (0.9)	7 (1.6)
Rec’d but no appointment scheduled	0 (0.0)	0 (0.0)	2 (0.5)	4 (0.9)	2 (0.5)	2 (0.5)
Missing	427 (100.0)	427 (100.0)	421 (98.6)	417 (97.7)	420 (98.4)	417 (97.7)
Patient screened within 24 h of admission (Tobacco)						
No	167 (39.1)	89 (20.8)	113 (26.5)	57 (13.3)	151 (35.4)	51 (11.9)
Yes	260 (60.9)	338 (79.2)	314 (73.5)	370 (86.7)	276 (64.6)	376 (88.1)
Missing	0 (0.0)	0 (0.0)	0 (0.0)	0 (0.0)	0 (0.0)	0 (0.0)
Patient screened positive (Tobacco)						
No	219 (51.3)	264 (61.8)	257 (60.2)	297 (69.6)	224 (52.5)	310 (72.6)
Yes	41 (9.6)	74 (17.3)	57 (13.3)	73 (17.1)	52 (12.2)	66 (15.5)
Missing	167 (39.1)	89 (20.8)	113 (26.5)	57 (13.3)	151 (35.4)	51 (11.9)
Patient received brief intervention (Tobacco)						
Received	7 (1.6)	28 (6.6)	19 (4.4)	31 (7.3)	17 (4.0)	33 (7.7)
Refused	0 (0.0)	14 (3.3)	6 (1.4)	7 (1.6)	4 (0.9)	7 (1.6)
Did not receive	0 (0.0)	8 (1.9)	8 (1.9)	5 (1.2)	4 (0.9)	5 (1.2)
Unable to determine	38 (8.9)	24 (5.6)	29 (6.8)	30 (7.0)	27 (6.3)	24 (5.6)
Contraindication	0 (0.0)	0 (0.0)	0 (0.0)	0 (0.0)	0 (0.0)	0 (0.0)
Missing	382 (89.5)	353 (82.7)	365 (85.5)	354 (82.9)	375 (87.8)	358 (83.8)
Patient received referral to treatment (Tobacco)						
Received	0 (0.0)	0 (0.0)	1 (0.2)	0 (0.0)	3 (0.7)	1 (0.2)
Refused	0 (0.0)	5 (1.2)	5 (1.2)	2 (0.5)	6 (1.4)	6 (1.4)
Did not receive	2 (0.5)	13 (3.0)	8 (1.9)	6 (1.4)	3 (0.7)	7 (1.6)
Unable to determine	40 (9.4)	45 (10.5)	39 (9.1)	53 (12.4)	34 (8.0)	41 (9.6)
Rec’d but no appointment scheduled	3 (0.7)	11 (2.6)	9 (2.1)	12 (2.8)	6 (1.4)	14 (3.3)
Missing	382 (89.5)	353 (82.7)	365 (85.5)	354 (82.9)	375 (87.8)	358 (83.8)

These outcome data were collected at three time points between January 2018 and May 2019: baseline, 10 months, and 16 months. At each data collection time point, 61 patient records at each unit (*n* = 854) were randomly selected by the health system using computerized random selection without human input to minimize selection bias [45]. Inclusion criteria were all records for adult (*≥* age 18) patients admitted and discharged from units selected for participation in this study within the last three months. A data abstraction tool for this information was developed based on the Joint Commission Quality Metrics for substance use and tobacco use. All site coordinators received training on data abstraction, and data were manually abstracted by site coordinators and entered in a fully de-identified format into a survey form on Qualtrics, a secure electronic data collection program. Some additional information not included in this study was also obtained through this method and is available in the deidentified dataset provided alongside this article. Investigators’ access to this limited, retrospectively compiled, and fully de-identified data in this manner was determined not to require individual patient consent as part of the IRB’s administrative review (see Study Design).

### Study size and statistical analysis for primary outcomes

The a priori sample size calculation was based on the approach outlined in the study protocol [12], which showed a need for 61 cases per cluster per time period to analyze absolute differences of 16% between study arms. Because the current study analyzed within-subjects changes as part of a cohort-based analysis, we infer that the actual power is greater than the planned power [46]. 

Levels of variable measurement are shown in Table 2 and were based on the extracted data structure. As steps in the SBIRT process progressed, missingness increased non-randomly. Specifically, all patients could in principle be screened, but only those who were screened could have been screened with a validated tool. Then, only those screened with a validated tool could have a positive or negative screening result (and so on). Considering this, we used the SPSS (IBM SPSS Statistics version 29) GENLINMIXED command to analyze numbers of screens (alcohol, drugs, and tobacco), numbers of screens using a validated tool (alcohol and drugs), and numbers of positive screens (tobacco). The confidence level was set at 0.95 but we report exact p-values [47]. The analyses used a binomial distribution with a logit link. In each model, time of data collection (i.e., baseline, 10-month, 16-month) was treated as a fixed effect (with intercept), and the hospital unit was treated as a random effect (with intercept), with the covariance type set as compound symmetry. We used Kenward-Roger adjusted degrees of freedom and report estimated marginal means for each time point as well as contrasts between baseline and each time point as odds ratios (OR, with 95% confidence intervals). We provide only descriptive statistics (frequency and percentage) for brief interventions and referral to treatment due to extremely high rates of missingness (predicated on event contingency for alcohol and drugs or incomplete documentation for tobacco).

## Results

### SBIRT documentation in EHR

At each study time point (baseline, 10-months, 16-months), 854 randomly selected patient records were successfully extracted (*n* = 61 per unit, with *n* = 14 units). Descriptive statistics about SBIRT endpoints by study group are presented in Table 2. Results from the within-group analyses of the SBIRT endpoints are presented in Table 3.

**Table 3:** Results of generalized linear mixed effects models, separated by group

	Baseline EMM (SE)	10-Month EMM (SE)	Exp(Coef.) (95% CI), *p*	16-Month EMM (SE)	Exp(Coef.) (95% CI), *p*
**Group 1**					
Patient screened within 24 h of admission (Alcohol)	0.623 (0.410)	0.749 (0.328)	1.81 (1.33–2.46), *p* <.001	0.653 (0.396)	1.14 (0.85–1.53), *p* =.396
Patient screened using a validated tool (Alcohol)	0.001 (0.326)	0.138 (47.173)	194.49 (57.33-659.76), *p* <.001	0.084 (30.428)	111.02 (33.00-373.53), *p* <.001
Patient screened within 24 h of admission (Drugs)	0.560 (0.439)	0.709 (0.368)	1.92 (1.43–2.57), *p* <.001	0.614 (0.422)	1.27 (0.94–1.66), *p* =.127
Patient screened using a validated tool (Drugs)	0.000 (0.124)	0.157 (51.946)	589.48 (78.61-4420.55), *p* <.001	0.093 (32.931)	322.26 (43.21-2403.42), *p* <.001
Patient screened within 24 h of admission (Tobacco)	0.628 (0.405)	0.768 (0.309)	1.96 (1.43–2.68), *p* <.001	0.671 (0.382)	1.21 (0.89–1.63), *p* =.220
Patient screened positive (Tobacco)	0.149 (0.180)	0.173 (0.202)	1.20 (0.76–1.89), *p* =.443	0.168 (0.198)	1.15 (0.72–1.84), *p* =.586
**Group 2**					
Patient screened within 24 h of admission (Alcohol)	0.844 (0.183)	0.935 (0.085)	2.66 (1.71–4.12), *p* <.001	0.955 (0.060)	3.92 (2.46–6.25), *p* <.001
Patient screened using a validated tool (Alcohol)	0.002 (532.978)	0.612 (65479.141)	813.93 (207.17-3197.77), *p* <.001	0.682 (59786.698)	1107.14 (280.05-4376.93), *p* <.001
Patient screened within 24 h of admission (Drugs)	0.661 (0.329)	0.901 (0.131)	4.68 (3.11–7.06), *p* <.001	0.925 (0.102)	6.31 (4.09–9.75), *p* <.001
Patient screened using a validated tool (Drugs)	0.002 (0.003)	0.372 (0.352)	327.46 (78.87-1359.55), *p* <.001	0.558 (0.372)	698.01 (166.67-2923.24), *p* <.001
Patient screened within 24 h of admission (Tobacco)	0.917 (0.103)	0.958 (0.055)	2.06 (1.29–3.29), *p* =.003	0.964 (0.048)	2.41 (1.49–3.91), *p* <.001
Patient screened positive (Tobacco)	0.221 (0.253)	0.198 (0.234)	0.87 (0.60–1.26), *p* =.470	0.174 (0.211)	0.74 (0.51–1.08), *p* =.124

**Fig. 2:**
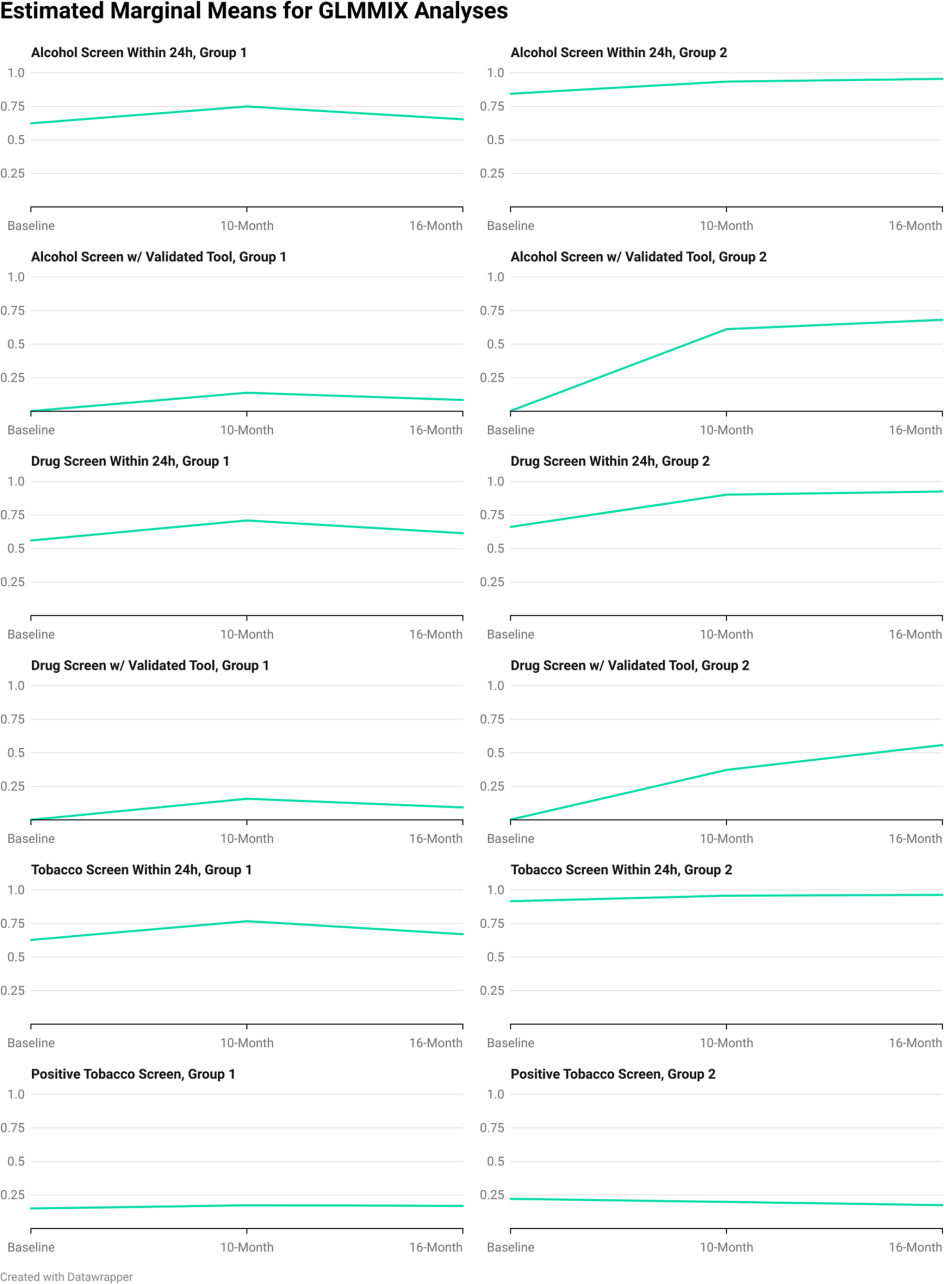
Panel graph visualizing key data from Table 3; estimated marginal means for GLMMIX analyses (Y) by data collection point (X)

For hospital groups 1 and 2, patients had 1.81 (1.33–2.46, *p* <.001) and 2.66 greater odds (1.71–4.12, *p* <.001), respectively, of being screened for alcohol within 24 h of admission at the 10-month project benchmark compared to baseline. Likewise, patients had 1.92 (1.43–2.57, *p* <.001) and 4.68 (3.11–7.06, *p* <.001) greater odds of being screened for drugs, and 1.96 (1.43–2.68, *p* <.001) and 2.06 (1.29–3.29, *p* =.003) greater odds of being screened for tobacco, respectively, within 24 h of admission at the 10-month project benchmark compared to baseline. For hospital group 2, patients also had greater odds of being screened for alcohol (OR = 3.92, 2.46–6.25, *p* <.001), drugs (OR = 6.31, 4.09–9.75, *p* <.001), and tobacco (OR = 2.41, 1.49–3.91, *p* <.001) within 24 h of admission at the 16-month benchmark compared to baseline, but for group 1, patient screening rates were statistically similar to baseline. Similarly, for both hospital groups, rates of positive tobacco screens did not appear to change significantly from baseline to either measurement period. Finally, statistical models for the use of a validated screening tool for alcohol or drugs converged less well due to the near absence of the use of such tools at baseline but still provided interpretable findings. For both hospital groups, and at both the 10-month and 16-month benchmarks, patients were hundreds of times more likely to be screened with a validated tool (*p* <.001 in all cases), reflecting a shift from near absence of such behaviors (at or under 1% prevalence) to prevalence rates from 24 to 56%. Graphs of the estimated marginal means (EMMs) for each of these models are provided as a panel in Fig. 2.

Very few patients screened positive for alcohol or drugs at any time point, despite the increases in the eligible population resulting from the increased use of validated screening tools. Consequently, very few patients received brief interventions or referrals to treatment for alcohol or drugs. In contrast, more patients were eligible for tobacco BI or RT (by virtue of screening positive). Some of these patients received brief interventions, but documentation was often unclear as to whether they received a brief intervention and was even more often unclear as to whether they received a referral to treatment.

## Discussion

This study examined the use of an implementation-focused SBIRT toolkit and TOT to facilitate initiation of SBIRT practices within a medical-surgical unit at 14 midwestern hospitals using a cohort design. Additional implementation information is available through qualitative interviews of nurses involved in the study [48]. 

The SBIRT intervention was associated with the initiation and sustained use of validated SBIRT screening tools for alcohol and drugs in medical-surgical units, and with short-term increases in overall alcohol, tobacco, and drug screening prevalence. Whether there was a long-term increase in overall screening rates was less clear. Before the intervention began (i.e., baseline), data from both groups of hospitals showed that more than half of patients were screened for alcohol, tobacco, or other drug use. However, consistent with prior research [35, 36], the alcohol and drug screenings rarely used validated tools at baseline. At the 10-month benchmark, overall rates of screening increased significantly, and the use of validated screening tools for alcohol and drugs had become more common. Then, at the 16-month benchmark, hospitals in group 1 saw overall screening prevalence return to baseline levels (no significant differences for T1 vs. T3), and the use of validated tools also declined for group 1 hospitals but remained significantly higher than at baseline. In contrast, hospitals in group 2 continued to see increased prevalence of screening and use of validated tools compared to baseline at both follow-up benchmarks.

Other studies of SBIRT-related screening rates in hospital settings have reported mixed results. Papers have variously suggested that completed screens were less prevalent in emergency care than in primary care [33], and occurred infrequently (8.4%) in emergency hospitals where site coordinators received a TOT and trained staff nurses [49], more than half the time (65%, including study participation refusal) among Polish emergency admissions [50], and nearly always (89-97%) when built into electronic triage procedures alongside training and support [51]. Screening rates for our site clusters ended up at 60-65% (group 1) and 87-88% (group 2) at the end of the study, alongside significant and encouraging increases in the use of validated screening tools from baseline values of 0.2-0.7% (group 1) and 0.5-1.2% (group 2) to 24-25% (group 1) and 56% (group 2).

At the same time, a core premise of validated screening tools is that they are more likely than direct questioning to accurately identify a patient’s level of risk. Therefore, while one should expect variations across different populations, regions, and other factors, rates of positive screening should generally be anchored in some way to established population-level rates. These could include national adult (18+) data, such as 6.3% past-month heavy alcohol use and 23.5% past-month binge drinking [52], combined alcohol/drug screening (22.4%) or pre-screening (18.4%) positive rates from clusters in a large cross-site evaluation [53], or data from individual studies (e.g., 21% hazardous drinking rates in an emergency department; [49] 9.7–10.8% unhealthy drinking rates in an integrated care SBIRT study [54]). Data from our study show comparatively lower absolute positive percentages (0.7–2.1% for alcohol and 1.4–2.3% for drugs), and relative percentages (i.e., with denominators reflecting only patients screened with a valid tool; 2.9–5.6% for alcohol and 4.4–7.2% for drugs, percentages not shown in Table 1).

We cannot know with certainty why rates of positivity in this study were lower than expected, though we do not think that it is especially plausible that these rates reflect true levels of risk for this patient population since a different hospital within the same region of Indiana reported positive screening rates for alcohol and drugs in line with other data sources [13, 55]. One possibility: many of the sites in our study adapted their SBIRT protocol such that all nurses were trained in screening while only one nurse (or a social worker) was trained to conduct brief interventions [42]. It is possible that nurses in some sites (those who were not trained to conduct brief interventions) were under-trained to administer the screenings with validity. Additional research on the necessary level of training to administer clinical questionnaires with validity might provide useful information. Another possibility, reflecting literature on SBIRT implementation barriers, is that alcohol and drug use remained stigmatized by some providers [56]. 

For alcohol and drugs, the low percentages of positive screenings made it infeasible to conduct reliable analyses for brief interventions and referrals to treatment. For tobacco, documentation of brief interventions and referrals was difficult to extract from the medical record due to inconsistent documentation. It is possible that brief interventions or referrals were provided but not documented or captured in the extraction process, but owing to this substantial unknown parameter, analyses would not be appropriate.

### Limitations

Despite our best efforts, this study did not adhere to the preregistered cluster randomized trial design and so was analyzed as a cohort. Within-subjects designs limit the ability to infer causality relative to randomized trials contrasting groups. A silver lining of this non-adherence is that it resulted from hospital unit leaders’ enthusiasm for SBIRT. Other limitations include the possibility of errors in data abstraction from the medical record, ongoing changes to the medical record system itself during the study period, and varying times to implementation by hospital (meaning that duration of operation prior to data extraction varied, sometimes by months). Generalizability is hampered because units selected to participate by the chief nurse officer may have differed from other medical-surgical units systematically (e.g., being perceived as the “most ready”). This study should be interpreted in light of these limitations and considered as part of a body of evidence, and not in isolation.

## Conclusions

This study adds evidence that an 8-hour SBIRT TOT session, SBIRT toolkit, and ongoing support for dissemination within hospital medical-surgical units may be associated with increased rates of asking about tobacco and screening for alcohol and drug use with validated tools, with some changes being sustained for a year or more. The strength of the evidence is lowered by changes after preregistration, including loss of the ability to conduct between-group comparisons for causal inference. However, the study is strengthened by a high degree of transparency, conservative interpretation, and open access to data and analytic materials. Important next steps likely include a nuanced study of how validated screening tools are used in primary care, as well as an expansion of the toolkit to facilitate more consistent documentation of outcomes (especially for tobacco).

## Data Availability

The raw data file received by RN and JA, along with annotations and statistical code, is available at the following link to the OSF repository: https://osf.io/vrxm4/?view_only=7bc8cb0da1df4125b52a90a9f7a0889d Individual hospital names were redacted to protect patient privacy but are not needed to replicate any analyses.

## Abbreviations

EMM: Estimated marginal mean
MI: Motivational interviewing
RN: Registered nurse
SBIRT: Screening, brief intervention, and referral to treatment
STROBE: Strengthening the reporting of observational studies in epidemiology
TOT: Training of trainers
USPSTF: United States preventive services task force
